# Integrating community health into primary care: two case studies from Barcelona's Raval neighborhood

**DOI:** 10.3389/fpubh.2025.1564009

**Published:** 2025-05-06

**Authors:** Francisco Ortega, Beatriu Bilbeny de Fortuny, Rocío Albuixech-García, Antonia Raya Tena, Josep Barceló-Prats, Marija Djurdjevic

**Affiliations:** ^1^Catalan Institution for Research and Advanced Studies (ICREA), Barcelona, Spain; ^2^Department of Philosophy, Anthropology and Social Work, University of Rovira i Virgili, Tarragona, Spain; ^3^Catalan Health Institute (ICS), Barcelona, Spain; ^4^Department of Nursing, University of Rovira i Virgili, Tarragona, Spain

**Keywords:** primary and community care integration, community-based health interventions, Filipino community, suicide prevention, structural competence, social and cultural determinants of health, adaptive healthcare

## Abstract

**Introduction:**

This study aims to generate evidence on healthcare practitioners' initiatives to integrate community health principles into primary care. Two case studies explore the co-design and co-development of tailored solutions to address the emerging health needs of vulnerable populations in the Raval neighborhood of Barcelona. The interventions aimed to improve access to healthcare services by establishing new care pathways adapted to the unique needs of migrant communities, while promoting inclusivity and equity in healthcare delivery.

**Method:**

An action-research approach was used during interventions conducted from December 2021 to March 2023 in Barcelona. This participatory iterative method included qualitative research to understand barriers hindering healthcare access and delivery; co-design of tailored training programmes focusing on structural and intercultural competences for both community members and HC practitioners; suicide prevention trainings for Filipino community representatives and PC providers; implementation of a community-based suicide prevention initiative; and evaluation of its effectiveness. Convenience and judgemental sampling engaged key stakeholders and influential figures from the Raval neighborhood. Sampling methods and R&I techniques are detailed in Case study 1 and Case Study 2.

**Results:**

Case Study 1 identified significant obstacles to healthcare access among immigrant populations, including linguistic, cultural, and discrimination-related barriers, stemming from inadequate administrative procedures and limited professional awareness of structural and social determinants of health. Case Study 2 highlighted the elevated suicide risk in Raval during the COVID-19 pandemic, leading to the co-design and implementation of suicide prevention training and the establishment of a sustainable, multi-stakeholder network of collaboration. Results from research and innovation activities are categorized in a table included in the text, with lessons learned discussed in the Discussion section.

**Conclusions:**

The findings underscore the critical role of primary care in identifying community needs and adapting services to meet the requirements of vulnerable populations through innovative approaches recommended by WHO and Medicus Mundi. Insights gained from these grassroots, bottom-up initiatives -driven by healthcare practitioners and conducted mostly during their free time- have been translated into actionable recommendations for policy and practice.

## 1 Introduction

### 1.1 Research reasons

The COVID-19 pandemic exposed significant limitations of top-down, one-size-fits-all public health measures, particularly their profound negative social impact on vulnerable populations. This crisis highlighted the urgent need for healthcare strategies that are more inclusive, adaptable, and responsive to diverse community needs (1, 2). Across the globe, alternative solutions emerged during the pandemic, driven by the necessity to address citizens' needs in innovative ways (3–5). These efforts revealed the vast potential of collaborations between primary care practitioners and community representatives. Extracting lessons from these initiatives offers valuable insights for rethinking and reorganizing primary care systems (6, 7).

Collaborative approaches are deemed essential for improving equitable access to healthcare services, for several reasons. Firstly, interdisciplinary collaboration brings together professionals from diverse fields, enabling a holistic understanding of the unique challenges faced by different populations. This diversity fosters searching for innovative and more effective solutions. Secondly, pooling resources through collaboration ensures the more efficient allocation of funds, equipment, and personnel. Thirdly, collaborative practices provide patients with consistent and coordinated care across services and providers, an important factor for managing chronic conditions and achieving continuity of care. Finally, integrating community representatives and local organizations into healthcare delivery extends care beyond medical interventions to include social and emotional support (8–12). This holistic approach makes healthcare more accessible, particularly for disadvantaged populations who often encounter barriers to accessing specialized services. Moreover, community-based care emphasizes preventive measures and health promotion, thereby reducing the burden on primary care systems while contributing to better population health outcomes (13–15).

Despite these clear benefits, the integration of primary and community care remains incomplete in many settings. Achieving greater connectivity requires significant changes in governance models and cultural shifts to promote collaborative approaches (9, 10). While some progress has been made, with certain European cities and neighborhoods building on the lessons of the COVID-19 pandemic, these examples are limited in scope (8, 11). There is a pressing need for more documented good practices to guide this transition.

Our study seeks to address this gap by presenting two examples of effective collaborative practices implemented during the health crisis in Catalonia (Spain). We present evidence of a successful local model that illustrates the feasibility and benefits of collaboration between primary and community care, examining also the underlying enablers that contribute to this collaboration. This local model not only offers practical insights into how to foster such collaborations but also serves as a catalyst for broader systemic changes in primary care.

### 1.2 Conceptual framework

#### 1.2.1 Community health and its principles

As stated by Goodman (16) “the meaning and strategic significance of *community health* remains challenging to fully define and to clearly distinguish from related areas of public health practice, community engagement, or other related community development activities.” However, scholars are recently embracing “a broader construct for *community health*” (17) considering its pluri-faceted nature, defining it as a “multidisciplinary, collaborative enterprise that uses public health science, evidence-based strategies” (16), and culturally appropriate approaches to engage and work with communities to optimize the health and quality of life of individuals within a geographically or socially defined population (18, 19).

The principles guiding community health emphasize inclusivity, equity, and collaboration. Key principles derived from recent scientific literature and political strategies and programmes include:

a) Cultural appropriateness: programmes must be tailored to the cultural contexts and needs of the communities they serve (17),
b) Community engagement: active involvement of community members in identifying needs, designing interventions, and evaluating outcomes is essential (20, 21),
c) Equity-driven approaches: equitable access to healthcare and resources across geographic, demographic, and social sectors (22),
d) Interdisciplinary collaboration: cooperation among health and social care workers, clinicians, policymakers, and community organizers to address complex health challenges,
e) Focus on social determinants: addressing factors such as socioeconomic status, housing, education, and environmental conditions is central to improving community health outcomes (23),
f) Long-term relationships: programmes should foster enduring partnerships beyond project timelines to ensure continuous improvement in community health systems,
g) Asset-based approaches: assessing community assets alongside needs allows for leveraging local strengths and capabilities in designing interventions (24).

#### 1.2.2 Primary care transformation

Community health, in the context of Primary Care innovation, can be defined as a collaborative approach that integrates population health management with person-centered clinical care, emphasizing community participation and equity-driven interventions (25). This framework combines three key theoretical pillars:

1) *Community-oriented Primary Care framework*

Community-Oriented Primary Care (COPC) is an approach to primary healthcare (PHC) that originated in South Africa and played a significant role in shaping the Declaration of Alma-Ata over four decades ago. In recent years, there has been a renewed focus on strengthening PHC systems and addressing critical knowledge gaps. COPC has proven to be an effective strategy globally, with notable success in places like Brazil and sub-Saharan African countries (26, 27). It integrates primary care with public health by addressing the health needs of defined populations and fostering collaboration between healthcare teams and local communities. Developed to bridge public health and primary care, COPC specifically aims to meet the unique needs of communities, particularly vulnerable populations, aligning with the World Health Organization's (WHO) principles that underscore primary healthcare as essential for achieving global health equity.

In Europe, initiatives such as the European Community Health Organizations (ECHO) have worked to advance COPC principles. The framework has been applied in diverse settings worldwide, including cities like Barcelona and Hong Kong (28, 29). These examples underscore COPC's potential to address community health challenges, while also highlighting barriers to implementation, such as insufficient government support, limited funding, and challenges in engaging healthcare professionals. In Catalonia, the COPC framework was formally integrated into the regional context through the development and publication of the National Strategy for Primary Care and Community Health (30).

2) *Integrated service delivery model*

The World Health Organization (WHO) global strategy on integrated people-centered health services (IPCHS) “is a call for a fundamental paradigm shift in the way health services are funded, managed and delivered” (31). It comprises the improvement of service design and delivery so that all people are able to access high quality health services that meet their needs and preferences. As highlighted by WHO: “This strategy calls for reforms to reorient health services, shifting away from fragmented supply-oriented models, toward health services that put people and communities at their center, and surrounds them with responsive services that are coordinated both within and beyond the health sector” (31).

In this regard, what was stated by C. Van Weel more than a decade ago is still valid “The future of primary care, and health care in general, will depend on how effectively primary practices achieve this community-oriented primary care approach and contribute to equity and social cohesion” (32, 33).

3) *Structural and Social determinants framework*

The social determinants of health (SDH) refer to non-medical factors that influence health outcomes, including “the conditions in which people are born, grow, live, work, and age” (34), as well as the broader forces and systems shaping these conditions. These determinants collectively affect health equity and access to healthcare influencing individuals' opportunities to achieve good health, their risk of illness, and their life expectancy. Structural determinants of health specifically encompass the social and political mechanisms that generate, sustain, and reinforce social hierarchies. These include factors such as the labor market, educational systems, political institutions and societal norms and values. “Among the contextual factors that most powerfully affect health are the welfare state and its redistributive policies (or the absence of such policies)” (35). Within the SDH framework, structural mechanisms are those that generate stratification and social class divisions within society, ultimately defining individuals' socioeconomic positions in hierarchies of power, prestige, and access to resources. This stratification results in social and structural determinants of health that are directly responsible “for health disparities, the differences in disease, injury, and opportunities for health witnessed by socially disadvantaged groups” (36).

Moreover, the World Health Organization states that the structural and social determinants of health (SSDH) together account for over half of population health outcomes, making them the single most significant contributors to individual health, surpassing the effects of genetics and personal behavior (34). In the context of Primary Care reform, addressing SDH involves integrating these factors into healthcare delivery systems to mitigate health inequities and improve overall population health outcomes. This requires moving beyond purely biomedical approaches to encompass the broader social, political, and structural determinants of health and wellbeing. According to C. Sobrino Armas, it is essential to avoid the pitfalls of medicalisation, overemphasizing individual responsibility and moralizing health issues, practices that risk detaching healthcare interventions from the social and political contexts that critically shape health outcomes (37).

To achieve this, healthcare and care workers must be equipped to understand how social determinants affect patients and communities. “Education of the health workforce is thus a key step to advancing action. Integration of the social determinants of health into education and training will prepare the workforce to adjust clinical practice and define appropriate public health programmes” (38).

Building on this conceptual framework, our study seeks to illustrate the practical application of community health principles through two Primary Care practitioners-led interventions conducted in Barcelona's Raval neighborhood, a district distinguished by its pronounced sociocultural diversity and marked socioeconomic challenges. Implemented during and after the COVID-19 pandemic, these interventions serve as exemplary models of community-oriented Primary Care.

### 1.3 Primary care and community health in Catalonia (Spain)

Primary Care (PC) in Catalonia has a strong tradition of community care. The roots of this tradition can be traced back to the enactment of the Primary Care Reform with Decree 84/1985 on March 21, 1985. Since that time, Catalan primary care has evolved from merely being the entry point to the healthcare system and the first level of curative assistance for the community, to also encompassing activities related to health promotion, disease prevention, rehabilitation, and psychosocial care, all integrated within a unified, holistic concept of health (39). Beyond the engagement of Catalan PC professionals in community health initiatives, such as promoting health education or organizing health fairs (40, 41), there has been substantial collaboration between them and social service practitioners to address the social determinants of health and deliver comprehensive care to the community. Furthermore, primary care professionals have been instrumental in supporting mutual aid networks to offer care and assistance to vulnerable populations (42). They have also participated in publichealth projects aimed at enhancing the overall health and wellbeing of the community, including vaccination campaigns and various health promotion activities.

There are numerous examples of initiatives based on collaboration between PC and community health initiatives that were implemented during several decades in Catalonia up until 2008. However, the economic crisis starting in 2009 constrained the development of these initiatives, relegating PC professionals to a predominantly care-oriented/assistant role (43). This shift weakened public health and community participation, as the social determinants of health ceased to be addressed collaboratively with the community. It was not until the implementation of the Health Plan 2015–2020, which included the National Strategy for Primary Care and Community Health (*Estratègia Nacional d'Atenció Primària i Salut Comunitària*, ENAPISC), that the community dimension of PC was revived in the region (44), Nonetheless, this renewed focus on the articulation of primary and community care lacked adequate resources and political commitment. Other limitations included minimal citizen participation and the scant integration of community health perspectives into the educational programmes and curricula of health degrees.

The onset of the COVID-19 pandemic in 2020 further stressed the Spanish healthcare system, particularly Primary Care. However, the pandemic did not alter the distribution of economic resources, exacerbating the underfunding of preventive and health promotion activities and concentrating public health expenditures on hospital and specialized services (45). Globally, the economic underinvestment in PC revealed itself starkly within weeks of the pandemic's onset, as many countries acknowledged their inability to “flatten the curve” through comprehensive public health measures. The reliance on top-down, one-size-fits-all measures to curb COVID-19 transmission had profound negative societal impacts, disproportionately affecting and exacerbating economic, racial, and gender inequalities (46). These challenges highlighted the need for context-sensitive, collaborative approaches in the design and implementation of health policies and interventions (1, 2). The participation of communities is essential because only community members possess the knowledge and lived experiences to inform public health professionals about their specific realities, perceptions of the pandemic, and locally feasible solutions for its containment (47).

## 2 Case study 1. Enhancing healthcare accessibility through participatory action: training and interventions for vulnerable populations in Raval

### 2.1 The Raval context

The Raval neighborhood of Barcelona, historically referred to as the *Barrio Chino*, is a central area of the city distinguished by notable sociocultural diversity. Located within the Ciutat Vella district, it houses 48,688 residents, 53.4% of whom were born outside the European Union. This population reflects over forty nationalities within a compact 1.1-square-kilometer area. The most prevalent nationalities, apart from Spanish, include Pakistani (16%), Filipino (16%), Bangladeshi (12%), Moroccan (6%), and Indian (5%), with an additional 8% comprising Italian nationals (Fundació Tot Raval). Compared to other areas of Barcelona, the Raval experiences considerably more challenging socioeconomic conditions (48–50). These disparities are evident in the neighborhood's streets, substandard housing and public infrastructure, characterized by an extensive number of precarious, poorly maintained housing units. Furthermore, the Raval has one of the highest unemployment rates in the city, nearing 50%. It also has one of the lowest average annual household incomes in Barcelona, at approximately €10,050 per household, in stark contrast to wealthier areas such as Tres Torres, where the average income reaches €30,284 (Statistical Institute of Catalonia, IDESCAT). Economic and employment insecurities have a profound impact on the majority of the population served by the neighborhood's Primary Healthcare Centers (*Centers d'Atenció Primària*, CAPs).

Historically, the Raval has been a service-oriented neighborhood. During the late 18^th^ and early 19^th^ centuries, it became one of Barcelona's first industrial zones, with the establishment of steam and textile factories requiring a substantial labor force, which contributed to a concentration of working-class residents. This working-class character facilitated the emergence of associations advocating for improvement of labor conditions, nurturing an activist spirit that continues to define the neighborhood (51). Over time, the Raval has consistently served as a point of entry for migrant workers, maintaining its role as a hub for those seeking employment (48, 52). Amid this historically, socioeconomically, and demographically intricate context, the Raval is home to a dynamic network of associations and entities dedicated to mitigating inequalities and promoting cultural diversity. These organizations work to integrate individuals at risk of social exclusion and address the precarious living conditions affecting a significant proportion of the neighborhood's residents. In the face of public administration's historical inability to meet community needs, grassroots movements have emerged, advocating for better living conditions through initiatives such as housing defense and the preservation of public spaces. Consequently, the Raval is an example of neighborhood self-organization and revindication of better public infrastructures for all. This neighborhood is a significant example of a community coming together to advocate for better public infrastructure and support for vulnerable groups (48, 53).

The organization called Fundació Tot Raval, established 20 years ago to improve social cohesion and the quality of life in the neighborhood, brings together around fifty social entities, educational institutions, cultural organizations, commercial associations, and individuals. The foundation's Community Health Programme, launched in September 2010 as part of the *Projecte d'Intervenció Comunitària Intercultural* aims to strengthen community efforts in addressing health determinants in the Raval neighborhood. Local collaboration of diverse stakeholders is fostered and funding from public and private resources secured, including public healthcare services [e.g., the Public Health Agency of Barcelona, a primary care center (CAP) Raval Sud, and the Immigrant Mental Health Service Programme], public social services and other service providers such as sports organizations, educational institutions, public facilities for older adults, and specific services for vulnerable groups. The programme aims to empower citizens and communities to take part in collaborative multi-stakeholder actions.

The Tot Raval Foundation's *Referents Comunitàries de Salut al Raval* initiative builds upon the principle that addressing health challenges requires the active involvement of the communities most affected by them. These *community health referrals* are volunteers with deep ties to the neighborhood, serving as intercultural mediators who navigate the intersection of public health systems and community needs. They work to identify barriers to accessing healthcare -such as linguistic, cultural, and systemic obstacles- and facilitate connections between residents and health promotion resources. By doing so, they not only advocate for equitable access but also foster trust and participation within a historically underserved population.

This initiative integrates diverse approaches, including capacity-building workshops for referrals, awareness raising campaigns targeting specific health issues, and participatory research activities to better understand the unique challenges faced by the neighborhood's multicultural population. These efforts aim to co-design culturally sensitive and contextually appropriate interventions alongside healthcare providers, policymakers, and community organizations.

Moreover, the programme seeks to embed health promotion within broader community dynamics, recognizing the interconnectedness of social determinants such as housing, employment, and education with health outcomes. For instance, referrals collaborate with social workers, educators, and housing advocates aiming to develop holistic strategies that could address the underlying causes of health disparities.

This way, *Referents Comunitàries de Salut al Raval* exemplifies a transformative model of community engagement that extends beyond traditional healthcare frameworks. By placing community members at the center of the process, the initiative addresses immediate health needs, fostering also long-term resilience and empowerment within the Raval's diverse populations. This collaborative, community-driven approach serves as a model for tackling health inequities in similarly complex urban settings.

### 2.2 Methods

In this intervention or action-research, a combination of research and innovation methods was used: qualitative research, co-design, and co-development of training workshops based on research findings (tailored to specific needs of differing stakeholders), and preliminary evaluation of the effectiveness of skills training for practitioners and community representatives, with a view to providing recommendations for decision-makers and informing policy.

In January and February 2023, the *Fundació Tot Raval* engaged in exploration of healthcare accessibility barriers in the neighborhood. One of the authors of this article (BBF), a healthcare practitioner, contributed to designing and implementing a qualitative study aimed at exploring the barriers vulnerable groups in the neighborhood face when accessing healthcare services. The study drew upon recommendations outlined in Medicus Mundi (54) report, *Barriers to the National Health System for Vulnerable Populations*. It aimed to gather narratives from citizens who took part in various health-related activities to understand their personal experiences with accessibility barriers. The findings were later used to guide a co-design of training workshops for local practitioners to reflect on how to address local challenges.

Convenience and judgemental sampling were employed to engage key referral mediating figures of the project *Referents Comunitàries de Salut*.

A total of nine in-depth interviews were conducted with migrant women from the neighborhood's four largest communities -Pakistani, Bangladeshi, Moroccan, and Filipino- aged 28 to 72 years, as well as with four physicians from the southern Raval Primary Care Centre, comprising two family doctors and two pediatricians aged 32 to 36 years. Purposeful sampling was employed to engage participants most relevant to the study. One criterion for participant selection was basic proficiency in Catalan or Spanish, as interviews with migrants were conducted without the use of translators or interpreters so as to maintain the authenticity of the narratives.

Ethical compliance and adherence to the General Data Protection Regulation (GDPR) were rigorously upheld, following the approval of the Ethics Commitee of the Fundació Tot Raval. Participants received detailed information about the purpose, methods, and potential risks of the research and voluntarily provided their informed consent. Their identities were anonymized, and confidentiality was maintained throughout the entire research process.

The research findings were presented at the February 2023 Plenary Session of the Raval's Community Health Committee. Its' Community Health Forum served as a platform for amplifying community voices and fostering dialogue between diverse stakeholders. Participants shared their lived experiences, identified persistent challenges, and furthermore co-created solutions aiming to enhance equity in healthcare service access. Finally, the insights gained during the forum informed a set of recommendations for the improvement of healthcare practices and policies within the Raval neighborhood.

The Quadruple Helix (QH) approach (55–58) was employed in this innovation action by involving four distinct types of stakeholders in participatory research and co-creation activities: healthcare practitioners, citizens, NGOs, and academics. This inclusive approach enabled the incorporation of perspectives from vulnerable groups into the co-development of solutions, thereby fostering innovation in service design and delivery. By centralizing the needs of vulnerable groups, the QH approach emphasized productive interactions based on the exchange of knowledge among the four key actors in the PC innovation system: science, policy, industry, and society (58).

### 2.3 Results

Based on narratives collected from both service users and healthcare providers, several barriers to healthcare access faced by vulnerable populations in Raval were identified. These barriers were categorized as linguistic, cultural, and those stemming from discriminatory practices and stigma.

#### 2.3.1 Linguistic barriers

Several service users highlighted that language barriers represent one of the most significant obstacles in healthcare centers. They stressed the urgent need for more cultural mediators to bridge these gaps. Additionally, the reliance on non-professional translators due to the shortage of mediators was also underscored:

“I explain that he cannot explain because he cannot say anything in Spanish, but they do not allow him to enter (the consultation room).” (S, Bangladesh)

“She asks if it is possible to have more mediators, as there aren't enough. More [mediators] are necessary because she has to call her brother every time she needs to go to the doctor.” (J, Pakistan)

At times, these limitations were so severe that users felt compelled to leave the system to receive adequate care:

“Now I have papers; my husband and my daughter also have papers. Once I have them, we will travel to India, and when we get there, I'll look for an eye specialist and book an appointment because here they don't understand me, and I think it's my fault - that I don't explain myself well, or I don't know...” (J, India)

Conversely, some users perceived it as discriminatory when professionals presumed they could not speak Spanish, treating them in a patronizing manner:

“When my daughters have bronchitis… I must take them to the pediatrician, and when I go there, they speak to me as if I've just arrived, as if I don't understand Spanish. They shout at me and say: ‘Do you understand? This is what you must do.' And I think, ‘Oh, my goodness!' Once, I even left (the consultation room) and told the girls at the desk: ‘Please, don't put me with her again.”' (H, Morocco)

Healthcare professionals, including doctors, also pointed out the burden of language barriers and their impact on clinical practice:

“Sometimes it's because you've had an awful afternoon, and then there's the language barrier - you just don't understand each other. They have several complaints in parallel … and you lose patience…” (B, doctor)

Professionals also mentioned the problem of the cutback of mediating services, which are crucial for bridging linguistic and cultural gaps between service users and service providers:

“For example, now there's a 10% decrease… five fewer hours for mediators. It's not moving toward better accessibility but the opposite: there is a problem of access.” (I, doctor)

#### 2.3.2 Cultural barriers

These barriers were the most frequently mentioned in interviews, encompassing both a lack of understanding of the healthcare system and insufficient consideration of cultural backgrounds. The cultural barriers are primarily concerned with the healthcare staff and could be addressed through specific intercultural training for professionals.

Regarding unfamiliarity with the Spanish medical system, it is worth noting that newcomers do not receive explanations on how the health system works upon arriving to the host country. Often, a lack of understanding about the organization among newcomers and rigidity of internal dynamics is interpreted by professionals as a lack of cooperation, disinterest, or misuse of the system. However, migrant interviewees highlighted significant conceptual differences between their countries' healthcare systems and that of Spain:

“I was with my brother in Accident & Emergency; we waited a long time, but there was no doctor. It was very strange because he was bleeding everywhere. I thought it was very dangerous, but there was no doctor.” (S, Bangladesh)

“*And in your country, do they attend to patients faster?” (Interviewer)* “Yes, it's faster, and the tests are better. More tests, less waiting, but you do have to pay for the visit.” (S, Bangladesh)

Another issue that arose was the rigidity of appointment and visit schedules, which clashes with the precarious employment situations of migrant healthcare service users and frequent lack of administrative documentation. This rigidity often hinders their ability to attend appointments:

“When I was at reception to book an appointment, it was hard for me to explain that I work from 9 to 10 and need an appointment before 9 or after 10 so that my boss doesn't get angry. It happens a lot… I didn't have papers, but I had to work…” (J, India)

On the other hand, cultural barriers manifest in the reluctance to discuss certain health topics, such as sexual or reproductive health:

“If I request an appointment, they ask: Why? but I don't agree with explaining everything at reception. If I say ‘Gynaecology', I don't want to explain what's wrong. In the waiting room, there are many people, even from my community. I can't say: ‘I have itching, I have discharge.' That must change, because it's not just me -everyone says the same-. Why do they ask this at reception?” (F, Bangladesh)

Another cultural trait of the way the service is used is a tendency to use emergency services directly instead of regularly visiting primary care professionals. This often led to late detection of serious health problems, making prevention and chronic health management difficult:

“(Patients) don't go to their GP (general practitioner); they go straight to emergency services. That's how it is in our country. Then you find cases where nothing can be done, the illness is already advanced. I've seen many cancer cases where it's already stage IV. They go, but there's no solution left… and then the family says: ‘We went there, and they didn't do anything.”' (F, Philippines)

#### 2.3.3 Barriers stemming from discrimination

One of the most notable topics to emerge during the qualitative inquiry was a discrimination of users by healthcare professionals, based on ethnicity or origin. It manifested in racist attitudes and inappropriate comments demonstrating significant lack of cultural sensitivity among practitioners.

When asked directly about their experiences of racist attitudes or comments, participants identified varying degrees of discrimination in different healthcare settings:

“In the early years, yes, I had language problems. But little by little, you start to communicate better with others… There are some who are racist, but not (all)… younger professionals today are good. But the older doctors, those from before… there's a stronger racism with them… but not with the younger ones.” (R, Philippines)

“She went to a doctor because her son had a fever during Covid. She used to make regular Covid tests, and it came back positive, so she told it to the doctor. And the doctor said: ‘No, no! You must leave, wash your hands!' And she felt like a monster - ‘Go out! This way! Stay home!”' (J, India)

Healthcare professionals also acknowledged instances of racism among their colleagues:

“*(Interviewer): Have you ever witnessed a situation of discrimination or racism… any comment… from a professional, a colleague, or in an emergency or hospital setting?”* “Many times! There's a range of what we call racism, from direct insults when the person isn't present to comments made when the person is right there but ‘won't understand me anyway.”' (I, doctor)

One user recounted a particularly humiliating incident of discrimination and mistreatment by a doctor. Following support from a local organization where she attended a language course, she filed a complaint supervised by the anti-discrimination office and received an apology. However, such responses are rare, often due to users' lack of knowledge about their rights or lack of access to support:

“It was a word, clearly… six or seven years ago. If I close my eyes, I see it clearly. If I go anywhere in the world… it's disrespectful, pointing a finger, saying, ‘Get out of here!' There's no explanation, just mistreatment. It's not respectful… Even if I don't understand the language, I do understand this behavior - it's the same in all languages. It's very ugly… I left crying, full of shame and anger because I couldn't explain myself well. Then I came here to Diàlegs, and Mercè asked: ‘What's wrong?'… I explained it to her. I didn't know about the Anti-Discrimination Office. I knew what discrimination was but didn't know where (the office) was. I went there, prepared the letter, and submitted it. Within two or three days, I received apology letter from the hospital and another one from the doctor.” (F, Bangladesh)

#### 2.3.4 Service innovation enablers and results

In terms of innovation, one significant outcome of the project was the *establishment of a multisectoral working group* dedicated to monitoring and addressing accessibility issues on an ongoing basis. Composed of representatives from community organizations, healthcare providers, and municipal agencies, this group aims to sustain the momentum generated by the participatory research, training sessions and forum. Regular meetings are being held to evaluate progress, share updates, and refine strategies based on emerging needs and continuous feedback from the community.

Another notable achievement and enabler of meaningful innovation was the *increased awareness* among healthcare professionals of the structural and cultural barriers faced by vulnerable populations. By engaging in self-reflection and dialogue, practitioners reported a greater understanding of how their own biases as well as systemic limitations impact citizen/patient experiences, especially among vulnerable groups (immigrants, older adults, women, etc.). This awareness has led to changes in service delivery, such as the adoption of culturally sensitive communication strategies and the implementation of simplified procedures to reduce bureaucratic hurdles.

The initiative also highlighted the critical role of *community health referrals* in bridging gaps between the public healthcare system and marginalized populations. Their active participation not only provided valuable insights into the specific needs of diverse community groups but also reinforced the importance of empowering community members as agents of change. The *collaborative, participatory approach* used in this intervention is now being considered for replication in other neighborhoods of Barcelona with similar demographic and socioeconomic challenges.

## 3 Case study 2. Community-driven suicide prevention intervention in the Filipino community of Raval Nord, Barcelona

### 3.1 Intervention context and premises

The second case study is a community-driven intervention performed in the Raval neighborhood focused on the Filipino community during the COVID-19 pandemic. Raval Nord hosts the largest Filipino community in Catalonia, which has been established in the area since the late 1970s (50) and is today being supported by a well-developed social network offering significant social support to Filipino citizens. Organizations such as the Intercultural and Social & Healthcare Mediation Team (EAMISS) represent the Filipino community within Raval's care system.

The COVID-19 pandemic severely impacted the over 10,000 Filipino citizens living in Barcelona, a community traditionally employed in hospitality, domestic service, and hotel cleaning (59). The prevalence of temporary contracts, restaurant closures, and informal work arrangements left many without income and unaware of how to seek assistance from public services, as community issues have historically been resolved internally (60). During the pandemic, EAMISS contacted the Raval Nord Primary Care Centre (CAP) to report the community's distress, exacerbated by job losses in the paralyzed hospitality sector. The organization sought collaboration with CAP in response to an alarming rise in suicides. Within a short period, the community reported five suicides and one attempt, none of which had been documented by the healthcare system (61). This community-based identification of an emerging health need formed the foundation of a joint suicide prevention initiative. The community request, combined with the alarming epidemiological reality, catalyzed a response from primary care professionals in Raval Nord.

This situation required a critical reassessment of how suicide risk was being addressed within the healthcare system. It highlighted significant gaps in training and preparedness, as most professionals had neither formal education on suicide prevention during their academic training nor practical experience in addressing it systematically. Practitioners recognized the urgency for immediate action and effective self-organization and began exploring the best ways to respond to the challenge at hand.

Initially, practitioners reviewed existing evidence to familiarize themselves with the phenomenon and then thoroughly investigated best practices in suicide prevention. Shortly thereafter, they initiated the intervention, ensuring that they were well-informed and prepared to implement strategies tailored to the Filipino community's specific needs. Their understanding of how to proceed was likely enhanced by the fact that several practitioners, both doctors and nurses, have recently completed doctoral and master's courses in Anthropology. To provide the most effective support and mitigate the rising incidence of suicides within the community, they opted to ground their actions in research evidence and follow the recommendations and guidelines of the World Health Organization (WHO). At the same time, they recognized the importance of collaboration. They were aware they could not achieve meaningful outcomes alone and that working with the local community was essential to strengthening the intervention and facilitating a comprehensive response to the crisis.

Research evidence shows the feasibility of suicide prevention within the primary care setting (62–64). Suicide is recognized as a complex and multifaceted phenomenon arising from an interplay of biological, psychosocial, and structural factors. Effective prevention strategies require an integrated etiological approach that includes addressing social determinants of health, which often cause despair and position suicide as a perceived solution (65). While professional and pharmacological interventions are critical for individuals with mental health conditions, broader interventions addressing the socio-environmental context are equally necessary (66, 67).

The Primary Care team recognized the importance of exploring narratives and interpretations of suicide within the Filipino community. Understanding how this community perceived and contextualized suicide was crucial for tailoring effective interventions. Community spaces, often centers of collective support and resilience, were identified as critical health assets because these spaces have the potential to act as protective factors by reducing isolation and offering constructive alternatives to the normalization of suicide as a solution to life challenges.

Moreover, the Primary Care team was aware of the importance of culturally sensitive approaches that respect and build upon the community's existing social frameworks. By engaging with leaders and active members of the Filipino community, such as the Guardians Brotherhood, this initiative leveraged pre-existing social structures to promote mental health and wellbeing. The collaborations between PC practitioners and community representatives were instrumental in co-developing a suicide prevention strategy that was both culturally resonant and clinically effective.

Finally, the team secured financial support for the intervention. The project proposal was submitted to a funding call by the Research Support Unit of the Catalan Institute of Health, which allocated protected hours for research action led by primary care professionals in Barcelona. This support facilitated the research, co-design and implementation of the activities.

The reflections and actions initiated through this intervention serve as a case study in bridging primary care with community health. They underline the need for integration of professional expertise with community knowledge to address complex health challenges. In the next section, we outline the specific methodologies and outcomes of this initiative, illustrating how community engagement and leadership can drive transformative health interventions.

### 3.2 Methods

Overall, action-research framework (68, 69) was used. Participatory research method (70) was applied to conduct qualitative research into Filipino community's attitudes toward suicide and its prevention, as well as to identify gaps in suicide risk management skills through two focus groups. This was later followed by innovation-driven activities including the co-design of tailored solutions (58), the implementation of co-designed training programmes to address the identified knowledge gaps, and the evaluation of these interventions using structured questionnaires.

The qualitative study spanned from May 2021 to March 2023, with data collection intervals from November 2021 to the final qualitative follow-up in March 2023. The sample consisted of nine Filipino community representatives, 6 men and 3 women aged from 41 to 75 years. For the quantitative data collection, referrals from guardians to the Primary Care Centre were considered. The number of diagnoses related to suicidal behavior in Ciutat Vella was analyzed using data from the Catalan national healthcare system, extracted on March 15, 2023. This data was then assessed to identify trends, patterns, and potential disparities in access to care among different age and gender groups. The analysis aimed to provide actionable insights for tailoring interventions to address the specific needs of vulnerable populations in the district during the COVID pandemic. Furthermore, the findings were cross-referenced with qualitative insights to ensure a comprehensive understanding of the issue and to inform the development of targeted suicide prevention action.

Ethical approval for the project was granted by the Jordi Gol Ethical Committee (Code: 21/207-P). Informed consent was obtained in the initial phase of the action-research (qualitative part) from all participants involved in focus group discussions (November 2021). For the quantitative inquiry, informed consent was obtained prior to the tests, including the pre-capacity-building questionnaire conducted in February 2022, the post-capacity-building questionnaire in March 2022 and the follow-up test in October 2022.

The action started establishing a dedicated working group (network of public administration, NGO, academia and communities representatives) in the Raval Nord neighborhood to address the suicide prevention among the Filipino community in Barcelona. Quadruple Helix approach (55–58) was used gathering healthcare practitioners, NGO managers, Filipino community representatives, and researchers. The group was coordinated by one of the authors of this article (RAG), a primary care practitioner, and operated under the framework of the project titled *Impact of a Community Intervention to Prevent Suicide in the Filipino Community in Barcelona* (61). The initiative sought to sensitize both community members and primary care professionals while improving access to healthcare services for individuals at risk of suicide. Therefore, their participation in co-design of targeted trainings was crucial. Co-creation paradigms (97) were appropriate for inter-disciplinary and inter-sectorial approaches in generating a new patient pathway.

The co-designed intervention consisted in two innovative actions: (1) *training community gatekeepers*, focused on equipping key members of the Filipino community with the knowledge and skills to identify and respond to individuals exhibiting signs of suicidal behavior, and (2) enhancing professional response, *addressing knowledge and skills gaps among primary care professionals*, with a specific focus on suicide risk management and the effective use of digital tools available within the healthcare system.

#### 3.2.1 Action 1 - Gatekeeper training

The intervention was co-designed to reach individuals in the Filipino community who might otherwise avoid healthcare services due to stigma or cultural taboos surrounding mental health and suicide. Gatekeeper training sessions followed the evidence-based QPR (Question, Persuade, Refer) approach, which teaches participants to identify warning signs, engage in supportive dialogue, and connect individuals at-risk to professional support and healthcare resources.

The core capacity-building intervention was provided by healthcare professionals between February and March 2022 comprising three sessions, each lasting 3 h. Pre- and post-training quantitative evaluations were conducted using structured questionnaires, with a follow-up evaluation 7 months later. This capacity-building intervention was a part of a multifaceted training strategy implemented over three consecutive years. In 2021, during the preparatory phase of the community gatekeeper intervention, online training sessions were conducted, emphasizing foundational knowledge. In 2022, the program transitioned to in-person clinical sessions led by professionals from the Community Mental Health Centre (CSMA) and members of the research team. Responding to persistent gaps in training, a gamified approach was introduced in 2023, utilizing an escape-room format to engage participants actively. Delivered over four sessions, this innovative methodology demonstrated significant improvements in professional confidence: self-reported competency in managing suicide risk rose from 28% before training to 84% afterward.

#### 3.2.2 Action 2 - Practitioner training

To better understand the needs of healthcare professionals, an anonymous survey was conducted among staff at the Raval Nord Primary Care Centre (CAP Raval Nord). The survey focused on identifying gaps in their knowledge, skills, and confidence related to suicide prevention. Key findings included the following acknowledgments: (a) *limited expertise in suicide risk assessment tools and national protocols*; many professionals were unaware of the tools embedded within the electronic clinical history system, ECAP (*Estació Cl*í*nica d'Atenció Primària*), which are crucial for activating the Suicide Risk Code as per the protocol established by the Catalan Government (71), and (b) *perceived insufficient training*; a significant proportion of respondents reported feeling inadequately prepared to manage suicide risk effectively, citing gaps in both theoretical knowledge and practical application of assessment tools and intervention strategies. Based on survey findings, targeted training sessions were developed and delivered to address these gaps. These sessions covered key topics, including (a) *use of ECAP tools* to activate the Suicide Risk Code; (b) application of structured *risk assessment protocols*; and (c) *best practices* in the clinical management of suicidal individuals.

Integrating these professional training sessions with the community-based gatekeeper training recommended by WHO, this intervention sought to foster a cohesive, multi-layered approach to suicide prevention, successfully bridging gaps between the healthcare system and the community.

The core of the intervention involved training community gatekeepers in suicide prevention, following World Health Organization (WHO) recommendations that acclaim this approach as both optimal and evidence-based (72, 73). Gatekeepers, defined as community members in strategic roles, were equipped with skills to identify warning signs of suicidal behavior, initiate supportive dialogue, and guide at-risk individuals to appropriate primary care resources. The goal was not professionalization but to empower gatekeepers with culturally sensitive, community-centered tools to address suicide risk, while fostering collective responsibility and strengthening community resilience.

In the case of the Filipino community, the intervention leveraged an existing structure of “guardians” drawn from the *Guardians Brotherhood tradition*, a fraternity model originating in the Philippines (74). Historically focused on addressing diverse community needs on a volunteer basis, this network was repurposed for suicide prevention. The Filipino community in Raval includes approximately 100 guardians, organized under a hierarchical structure where each leader oversees ten guardians. Nine leaders (three women and six men; average age: 52.25 years) participated in the training conducted between May and October 2021. This period coincided with pandemic-related job losses in the hospitality sector and the rollout of COVID-19 vaccinations, which alleviated some demands on PHC professionals.

QPR methodology (Question, Persuade, Refer) (75, 76) was employed for gatekeeper training, a widely recognized evidence-based strategy for suicide prevention. This model emphasizes three practical steps: (1) *Question*: Directly inquiring about suicidal thoughts or intentions, (2) *Persuade*: Encouraging individuals to accept support, and (3) *Refer:* Connecting individuals to appropriate community or professional resources.

The training team was composed of a multidisciplinary group from the Raval Nord Primary Care Centre, including: two nurses (specializing in family and community care, and mental health), two resident nurses specializing in family and community care, a nursing care assistant with expertise in psychopedagogy, an administrative staff member, a psychologist specializing in emotional wellbeing, and a resident doctor specializing in family and community care. To ensure alignment with best practices, the instructors themselves underwent QPR training, often outside of working hours.

### 3.3 Results

Focus groups revealed that participants shared common beliefs about suicide, including its perceived unpredictability and its strong association with mental health disorders. Cultural factors such as religious beliefs and the stigma surrounding mental illness emerged as critical themes, requiring special attention during gatekeeper training.

After the training intervention provided by healthcare professionals from the PCC Raval Nord, *immediate improvements* were demonstrated in participants' knowledge of suicide prevention and the QPR (Question, Persuade, Refer) method for effective derivation of citizens to relevant health and social care services. While some decline in knowledge retention was observed after seven months, participants reported sustained and increasing confidence in their ability to support individuals at risk.

Participants highly valued the training, with one participant, Emily Silang, a business owner and member of the Filipino Guardians Brotherhood, emphasizing its significance in identifying early warning signs of suicide within the community. Silang highlighted the *economic challenges* faced by the community as a contributing factor to increased suicide risk (61). She also expressed her intention to disseminate the knowledge gained through Filipino community associations. Moreover, participants proposed reinforcement training sessions to maintain knowledge and confidence and suggested offering *sessions in Tagalog to increase accessibility* and reach a broader audience. The initiative received the endorsement of the Filipino Consulate in Barcelona, which expanded its visibility among other Filipino associations. However, concerns about potential stigmatization limited broader participation.

By 2023, the easing of pandemic restrictions enabled the Filipino community and healthcare professionals to re-assume regular activities. This shift led to diminished collaboration and deceleration of multi-stakeholder processes. However, media coverage of the intervention drew attention to its positive impact, reporting on the epidemiological realities and the community's preventive efforts without sensationalism (61). This exposure stimulated interest from additional community organizations, leading to new *collaborations between the Primary Care Center (CAP Raval Nord) and local stakeholders*.

As a result of these new partnerships, *novel capacity-building initiatives* emerged, such as *Training on Emotional Well-Being and the Filipino Neighborhood for Ciutat Vella's Community Agents* initiative, marking a significant step in expanding the scope of community-based mental health interventions. The programme not only addressed immediate challenges but also strengthened the infrastructure for long-term support. Participants acquired skills to identify signs of emotional distress and became familiar with navigating the complexities of local health and community systems. This knowledge empowered them to act as intermediaries, linking individuals in need with appropriate services. Filipino community organizations played a pivotal role in ensuring strong participation in the training sessions. Their involvement also helped bridge cultural and systemic gaps between the community and healthcare providers. However, some challenges persisted. Participants highlighted concerns about the lengthy waiting times for accessing mental health services, which could delay critical interventions. Despite these barriers, the initiative successfully fostered collaboration among diverse stakeholders and increased awareness of mental health resources within the community.

As an effect of the intervention, following the training, a group of Filipino-origin psychologists took the *initiative to form an association* aimed at addressing mental health challenges specific to their community. Plans were made to integrate this group into the Ciutat Vella District Mental Health Table (Taula de Salut Mental del Districte de Ciutat Vella), further enriching the collaborative framework. This development underscores the initiative's ripple effect, inspiring community-driven actions to complement formal healthcare efforts.

One of the main results of the intervention is a *sustained impact on suicide prevention*. Since the initial response to the community's request in 2021, the Primary Care Centre (CAP Raval Nord) has maintained its commitment to suicide prevention and mental health promotion. *Regular training and sensitization efforts for primary care professionals* have continued, ensuring that the healthcare team remains equipped to address suicidal behavior effectively. On the other hand, evaluation results show the effectiveness of collaborative community-driven interventions: both quantitative and qualitative data indicate measurable improvements, and the detection of suicidal behavior within the Raval Nord Primary Care Team now surpasses that of other teams in the Ciutat Vella district. Referrals, initiated by community gatekeepers, have been consistently linked to appropriate health and social services underscoring the effectiveness of this integrated approach.

## 4 Discussion

Previous studies in this domain have addressed numerous community-based suicide prevention programmes in primary healthcare settings across urban areas in Iran (77), Brazil (78–80), China (81), Germany (82), amongst other, as well as rural suicide awareness and intervention initiatives in regions like Japan (83), Tasmania (Australia) (84), or India (85). Similarly, preceding studies have vastly explored educational interventions aimed at enhancing intercultural and structural competencies among primary care professionals in various global contexts (86–89).

However, most of these studies focus on interventions that are integrated into national, regional, or municipal strategies, typically guided by public administration managers as part of structured plans to enhance healthcare services and adapt them to increased immigration flows. In contrast to these large-scale, top-down initiatives, this study examines two small, spontaneous, bottom-up actions led by primary care practitioners, largely during their free-time. These grassroots interventions arose in response to issues directly raised by vulnerable groups during the unique challenges of the COVID-19 pandemic. Led by nurses and physicians, these efforts are particularly noteworthy as they highlight the awareness, commitment, and adaptability among certain Spanish healthcare professionals. Beyond meeting the immediate needs of their communities, these healthcare practitioners also emerge as voluntary leaders and agents of systemic change.

The synthesized results of the interventions carried out in Barcelona, presented in Case Study 1 and Case Study 2 and outlined in Table 1, aim to identify the shared elements and key components of these initiatives. Through a systematic review of the outcomes, this study seeks to extract insights and lessons learned from the research and innovation processes undertaken in the multicultural Raval neighborhood.

**Table 1:** Over view of results.

**Action-research in Raval**	**Case study 1**	**Case study 2**
**Research**	**Results**	**Results**
Qualitative research on **barriers** influencing access to healthcare services by vulnerable populations (immigrants)	•Linguistic barriers identified •Cultural barriers identified •Stigma and discrimination practices identified	•Knowledge/skills barriers among vulnerable populations identified •Knowledge/skills barriers among healthcare practitioners identified •Stigma surrounding mental illness acknowledged
Literature research on **enablers** influencing access to healthcare services by vulnerable groups	•Effective communication between healthcare workers and vulnerable groups •Partnership established for collaborative actions between practitioners and community members •Patient-centered perspective in service re-design	•Effective communication between healthcare workers and vulnerable groups •Partnership established for collaborative actions between practitioners and community members •Patient-centered perspective in service re-design
**Action**	**Results**	**Results**
**Co-design** of capacity-building and awareness raising interventions	•Skills training for practitioners to acquire cross-cultural competences •Skills training for practitioners to acquire structural competences (social determinants of health)	•Capacity-building for practitioners to acquire expertise in suicide risk management •Capacity-building for gatekeepers (Filipino community members) to acquire skills for early detection of warning signs and derivation to PCC
**Collaborative actions** based on Primary and Community care integration (co-design of solutions for effective access to healthcare services for vulnerable populations)	Qualitative: •Increased awareness (cultural humility, structural sensitivity) among practitioners •Understanding and trust built (negative health outcomes and lifestyles are shaped by larger socio-economic, cultural, political and economic forces) among healthcare workers •Accountability for building more equitable health system	Qualitative: •Increased awareness among vulnerable groups (cultural shift toward open and stigma-free discussions about suicide) •Trust and partnership built among Filipino community organizations and healthcare practitioners, creating safe communal spaces •Service access improved (new patient journey) •Further training co-designed in emotional wellbeing to sustain suicide prevention efforts •Regular training and sensitization for PC practitioners to address suicidal behavior
	Quantitative •No quantitative data was collected	Quantitative •Network of 10 Filipino community referrals (gatekeepers) established •Competency in managing suicide risk rose from 28% before training to 84% afterwards

In the action-research activities, the empirical data gathered through qualitative research resulted, firstly, in the *identification of barriers* to accessing healthcare services in both case studies. Detected obstacles can be broadly categorized into three primary domains: (1) barriers stemming from an immigrant condition of community members (linguistic difficulties, social discrimination, auto-stigmatization, real or perceived lack of rights, and amongst other); (2) barriers resulting from “traditional” way of doing healthcare (limited cultural sensitivity, discriminatory attitudes toward immigrants); and (3) systemic barriers, stemming from public administration procedures and management practices rooted in structural inefficiencies. These two case studies created valuable evidence on specific *local obstacles* that hinder access to healthcare services to vulnerable groups causing several negative effects on society, such as the increase in health disparity, reduced work productivity, higher rates of mortality and social strain.

Secondly, healthcare practitioners from the PCC in Raval (Barcelona) took an active role in exploring how these barriers could be mitigated. From the literature and desk research they extracted valuable knowledge on factors that facilitate access to healthcare services (*identification of enablers*) by vulnerable populations, based on the engagement of their community representatives in the joint search for solutions (21, 31, 54, 72, 73, 90). In the Case Study 2, for the specific challenge -the increase in suicide rates during the COVID-19 pandemic among Filipino community- practitioners utilized the WHO guidelines for recommended and evidence-based suicide prevention interventions carried out successfully in other parts of the world. They decided to follow and adapt those guidelines to the specific context of the Raval neighborhood and the Filipino community that reported about sudden suicide cases asking for help. Enablers of the mitigation action that was co-organized by healthcare workers and community representatives were the following: the communication channels opened, networks created comprising PCC, NGOs and local enterprises, as well as partnerships established between professionals and community representatives for continuous collaboration and joint development and implementation of solutions.

Thirdly, the PCC practitioners organized appropriate *skills training*s for both practitioners and community members (see the Table 1) decidedly addressing the identified barriers. The trainings ranged from sensitivity building to capacity building, preparing differing societal actors to overcome local obstacles and address citizens' real needs. Lastly, after building a network for collaboration of public institutions and communities, and acquiring a necessary level of awareness and skills, the Raval PCC practitioners initiated community-based interventions. The Case Study 2 shows that they succeeded in improving access to health and social care services by vulnerable populations at risk of suicide and stopped the increase in suicide rates.

### 4.1 Lessons learned in this action-research

Several lessons have been learned during the mentioned action-research processes, as follows:

#### 4.1.1 Lesson 1

Healthcare professionals often have limited cultural sensitivity, and in stressful situations caused by work overload, they may engage in discriminatory attitudes that hinder the access to healthcare services of vulnerable immigrant groups. This kind of practitioner-related barrier significantly undermine the quality and inclusiveness of care, but they can be effectively mitigated through *comprehensive training programmes for healthcare practitioners*. These programmes should focus on developing cross-cultural and structural competencies to equip professionals with the skills to navigate diverse patient needs addressing also root causes of social inequities (91–93).

#### 4.1.2 Lesson 2

Vulnerable populations (immigrants) show to be underinformed about their rights, have linguistic difficulties and suffer a cultural shock, stigma, and self-stigmatization. Their understanding of Catalan socio-cultural codes, language competences, and skills to navigate healthcare system can be effectively improved when receiving help from their peers (communities). In the Case Study 2, the comparative analysis of pre- and post-intervention results underscores a significant improvement in Filipino community members' understanding of their situation. One of the most significative results is a cultural shift toward open and stigma-free discussions about suicide, meaning that safe communal spaces were created during the educational intervention that are critical for suicide prevention. The lesson learned is that fostering communication and collaborative action of communities and practitioners, and *training community representatives in “gatekeeping”* they are properly empowered *to provide peer support* and promote cultural and social integration in the Catalan society.

#### 4.1.3 Lesson 3

Systemic barriers stemming from public administration procedures are rooted in structural inefficiencies. But national healthcare systems are today undergoing significant transformation and experimental interventions are promoted to create evidence on effective novel practices, new roles, and solutions. It is further required to analyse innovative solutions to extract lessons learned that can *guide the development of targeted public policies* aimed at eliminating inequities and enhancing accessibility to healthcare services for all.

#### 4.1.4 Lesson 4

However, it is not enough to organize targeted occasional skills training or capacity building. The observed decline in knowledge retention detected in these case studies during follow-up activities mirrors findings from similar studies (73, 90, 94), emphasizing the *need for continuous reinforcement of training* to maintain the interventions' long-term benefits. This highlights a key lesson for public health policies: that training programmes must be designed not only for initial impact but also for sustainability through periodic refresher sessions.

#### 4.1.5 Lesson 5

*A community-based approach is essential for new healthcare policy*, as it introduces critical knowledge and awareness required to address health inequalities and challenges. Strategies and policies should be developed that foster the involvement of communities in healthcare service design and delivery. As stated by experts, active involvement of communities in decision-making processes fosters trust and empowers communities and citizens to influence social determinants of health, thereby reducing disparities (37). Policies that enable building and maintaining robust public health ecosystems that integrate communities is especially important in neighborhoods like the Raval, where discrimination and inequality are prevalent. Community groups possess intimate knowledge of the specific health challenges and needs within their area, making their input invaluable for designing effective interventions.

#### 4.1.6 Lesson 6

*Participatory methods* of service creation and delivery show to be particularly impactful. The evidence from these case studies exemplifies how community health interventions can effectively address complex socioeconomic and cultural challenges through *joint work of neighbors, healthcare providers, and community organizations*. It demonstrates the transformative potential of community-based interventions when grounded in participatory methods and intercultural collaboration.

#### 4.1.7 Lesson 7

Case studies 1 and 2 demonstrate the *adaptability of Primary Care* and its capacity to creatively react to emerging health challenges. Primary Care practitioners showed capability and responsibility to approach disease prevention incorporating (a) education of both practitioners and community representatives in social determinants of health and (b) community-driven initiatives as complementary strategies. By addressing accessibility barriers through targeted educational actions that foster dialogue and build intercultural and structural competencies these interventions have laid the foundation for more inclusive and equitable healthcare systems that genuinely reflect the needs and aspirations of the communities they serve.

Our multidisciplinary team of healthcare professionals and researchers, with a shared focus on Primary Care innovation and community integration, reflected on factors that influenced Raval's collaborative success. We identified three key factors that shaped the above mentioned good practices in the Catalan context: (1) the high level of awareness among primary care professionals of the cultural and structural factors affecting immigrant populations' healthcare access—largely informed by higher anthropological education of several practitioners; (2) the tradition of “commoning” (peer governance and provisioning) with pre-existing communitarian structures within the Filipino Brotherhood; and (3) the historical legacy of active collaboration between primary and community care, a hallmark of the Catalan healthcare system.

Following the lessons learnt from these interventions, we underline the importance of cultivating culturally sensitive, community-rooted, and collaborative approaches. These approaches not only address the immediate needs of vulnerable populations but also lay the foundation for sustainable, equitable healthcare systems adaptable to diverse global contexts.

### 4.2 Future research directions and upscale

To align more closely with global and local healthcare innovation strategies, future initiatives should prioritize scaling and adapting the described models to other vulnerable communities, while respecting their unique cultural and structural contexts. The described actions could be upscaled among differing immigrant communities, in other Barcelona districts as well as in cities worldwide.

Regarding future research directions, investigating the long-term impacts of such interventions—particularly their potential to foster self-sustaining networks of care—will be essential. Equally valuable would be research into the roles of education and technology in promoting effective collaboration, along with innovative methods for reinforcing skills training and capacity building. Furthermore, *context-specific studies* guided by the Realist Evaluation framework could help identify what works, for whom, and why, thereby offering valuable insights for optimizing collaborative practices (95). Finally, qualitative research on healthcare service innovation should be complemented with quantitative data to reveal the cost-effectiveness and impact on vulnerable groups' health showing the practical benefits of these interventions that are crucial for informing policies.

## 5 Conclusions

This study, based on combined qualitative and quantitative findings, provided a robust evidence base to inform the co-design and implementation of tailored community-based interventions. It sought to establish a framework for replicating participatory methods in other contexts. The two community-based interventions described highlight the vital role of Primary Care in identifying community needs and reorganizing services to address them effectively.

Attempting to integrate community health principles into primary care, the case studies exposed provide several key insights and recommendations for decision-makers and healthcare practitioners.

### 5.1 Recommendations

In relation to the extracted lessons learned, the recommendations for policy and practice are the following:

1) Promote *regular training of healthcare practitioners* in structural, social and cultural determinants of health, person-centered service creation and delivery, and participatory service co-design methodologies.
2) Encourage *regular consultation with community representatives* to identify the unmet health and social care needs of vulnerable groups, including mental health, either through collaboration with existing community organizations or by engaging highly motivated individuals.
3) *Actively involve community members in the re-design of existing services* to ensure their needs are accurately addressed, and in co-design of new protocols and patient pathways to enhance relevance.
4) Identify *good practices* of service innovation from across the globe that can be scaled within the Catalan integrated care context.
5) Promote *collaborative community-driven interventions* by insuring healthcare practitioners are equipped with time and resources needed to focus on developing improved services.
6) Strengthen *academia-industry partnerships* to advance joint research and innovation activities, prioritizing interdisciplinary collaboration.
7) Empower *primary care practitioners to embrace the role of change leaders* by actively involving communities in service innovation (provide them with the necessary tools and training to engage stakeholders, ensuring that community voices are integrated in healthcare reform).

### 5.2 Policy implications

The evidence presented in this article aligns closely with the overarching objectives of preventive health policies and public sector innovation strategies.

Firstly, the findings from Case Study 2 support the World Health Organization's (WHO) Global Action Plan for Suicide Prevention, highlighting gatekeeper training as an essential strategy to reduce suicide rates. These interventions also resonate with European Union priorities in preventive healthcare, particularly in empowering citizens and fostering resilience through community participation. Furthermore, their capacity to enhance awareness and accountability aligns with initiatives aimed at promoting self-care among citizens. By engaging community members as active participants in their own health, the interventions exemplify the principles of health literacy and community empowerment—core pillars of global health promotion frameworks such as the Ottawa Charter for Health Promotion. In addition, these innovative efforts align with the European Commission's Horizon Europe Missions, contributing to a healthier Europe through addressing the challenge of creating citizen-centered solutions. The interventions presented exemplify practical applications of the Health in All Policies (HiAP) framework, addressing social determinants of health to promote equitable health outcomes.

Moreover, the findings hold relevance for public sector innovation strategies (96). Policies that encourage participatory governance and intersectoral collaboration (38) could find practical applications in the case studies discussed. The results demonstrate how incorporating local knowledge and cultural sensitivity into healthcare strategies can reinforce the relevance and effectiveness of public health programmes. The synergies created by bridging community associations, primary care teams, local organizations, and researchers provide an exemplary model for healthcare innovation endeavors. Additionally, the case studies offer models for developing sustainable health assets within communities. Initiatives like the *Filipino Community Emotional Well-being Commission* established post-intervention illustrate how such efforts can drive systemic change.

Specifically, the findings from this study advance the promotion of Community-Oriented Primary Care (COPC) principles both across Europe and globally. By showcasing the ability of COPC to address diverse community health needs and highlighting the challenges encountered during implementation, this study provides valuable insights into the practical application of these principles. These examples of good practices significantly enrich the resources available to the European Community Health Organizations (ECHO) network and serve as valuable contributions to the Catalan National Strategy for Primary Care and Community Health (44).

In conclusion, to expand upon successful practices, future policies should focus on sustaining community engagement through participatory governance models, investing in training programmes that enhance the intercultural and structural competencies of healthcare professionals, developing comprehensive suicide prevention strategies that integrate community health assets, and allocating dedicated resources to support community health initiatives as integral components of Primary Care.

The interventions in Raval exemplify how integrating community health into Primary Care can be a response to immediate challenges, but also a transformative strategy for creating equitable and sustainable healthcare systems.

## Data Availability

The original contributions presented in the study are included in the article/supplementary material, further inquiries can be directed to the corresponding author.
